# Supplementary material for: MolAI: A Deep Learning Framework for Data-Driven Molecular Descriptor Generation and Advanced Drug Discovery Applications
Source: J Chem Inf Model. 2025 Sep 15;65(19):9892–909. doi: 10.1021/acs.jcim.5c00491 (PMC12529771; doi:10.1021/acs.jcim.5c00491)
Supplement: Supplementary file 1 [file ci5c00491_si_001.pdf]

Supplementary Material for

MolAI: A Deep Learning Framework for Data-Driven Molecular Descriptor Generation and Advanced Drug Discovery Applications

Sayyed Jalil Mahdizadeh and Leif A. Eriksson*

***Corresponding Author**

Email: leif.eriksson@chem.gu.se

This PDF file includes:

Figure S1. The models architectures S2

Table S1. Summary of datasets used in the study S2

Figure S2. Examples of compound interpolations S3

Figure S3. Distribution of the latent vectors, and de novo compound generation S5

Table S2. Pearson correlation coefficients and p-values for molecular descriptors S6

Figure S4. Weights assigned to each PCA component for predicting the molecular descriptors S6

Figure S5. Examples of protonation states predicted using iLP S7

Table S3. Datasets used for ADMET features predictions S8

Table S4. Summary of iADMET performance metrics using MolAI descriptors S9

Figure S6. ADMET feature predictions based on ChemBERTa-77M-MTR descriptors S10

Table S5. Summary of iADMET performance metrics using ChemBERTa-77M-MTR descriptors S11

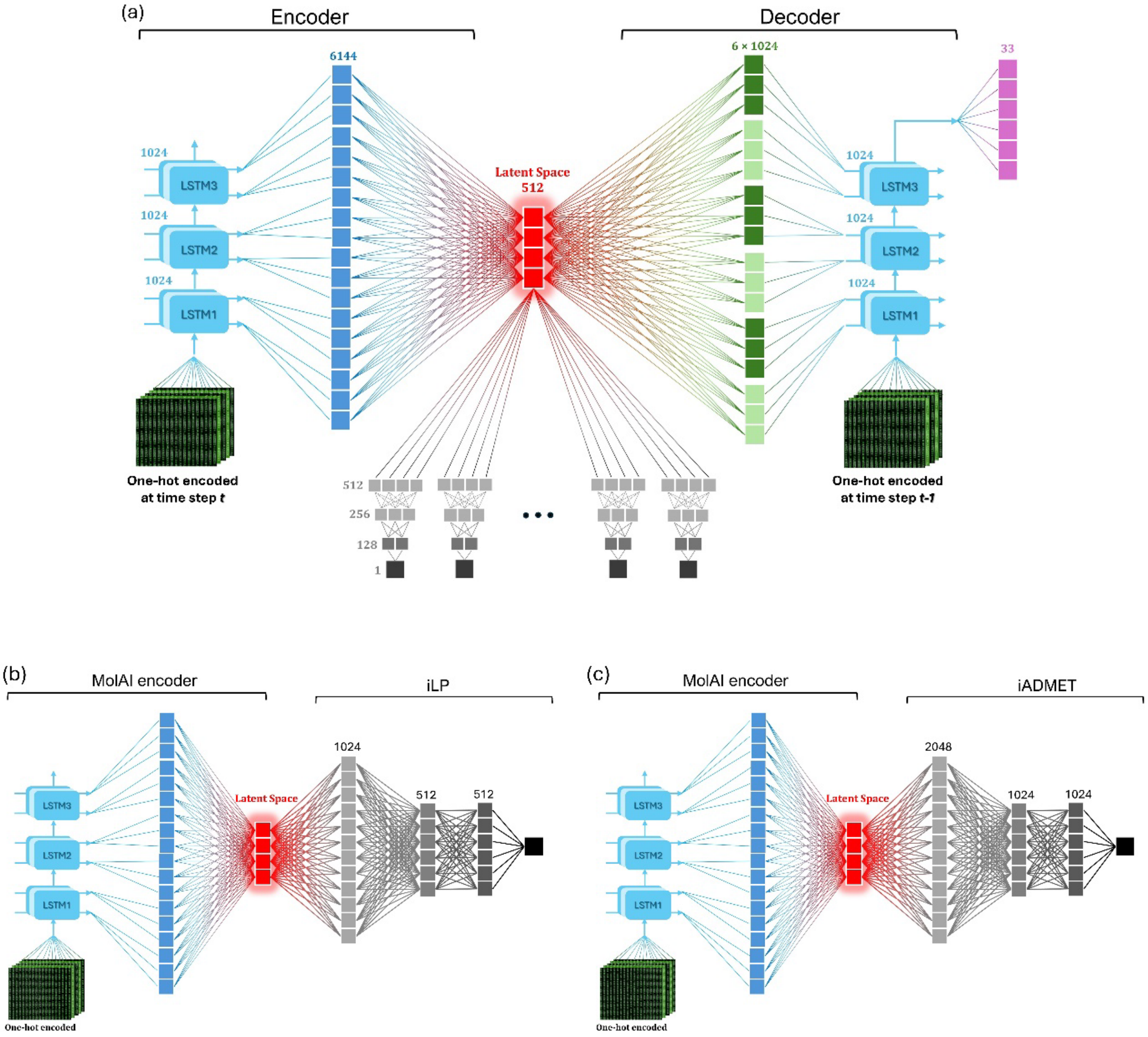

Fig. S1. The model architecture for **(a)** MolAI, **(b)** iLP, and **(c)** iADMET.

Table S1. Summary of datasets used in this study.

Dataset	Total size	Train/Validation	Source	Access link
**MolAI - training**	221M	10/2	ZINC15 + PubChem	https://zinc15.docking.org<br>https://pubchem.ncbi.nlm.nih.gov
**MolAI - test1**	1M	-	ZINC22	https://cartblanche22.docking.org
**MolAI - test2**	200k	-	MolPort	https://www.molport.com
**iLP - training**	10M	10/2	ZINC22	https://cartblanche22.docking.org
**iLP - test1**	10k	-	Enamine Real	https://enamine.net
**iLP - test2**	24k	-	Hinge@Enamine	https://enamine.net
**iLP - test3**	10k	-	ZINC22	https://cartblanche22.docking.org
**iLP - test4**	10k	-	Generative AI	https://www.anyolabs.com
**iLP - test5**	10k	-	Generative AI	https://www.anyolabs.com
**iLP - test6**	10k	-	Generative AI	https://www.anyolabs.com
**iADMET-training**	See Table S3	10/2	Therapeutics Data Commons	https://tdcommons.ai

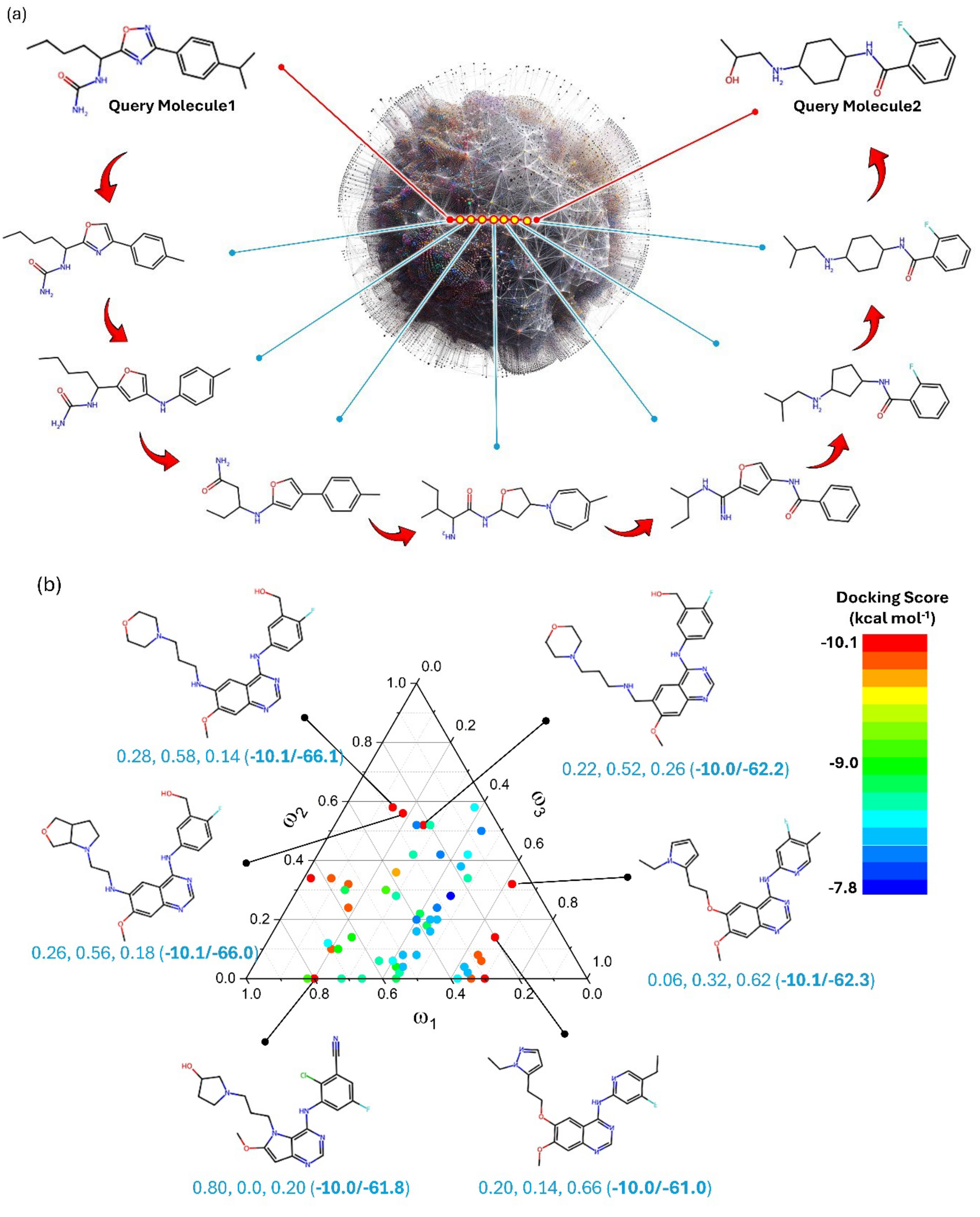

Fig. S2. (a) Example of new compounds generated using bi-molecular interpolation by scanning through the latent space between two query molecules. **(b)** A tri-molecular interpolation for Top1, Top5, and Top7 Gefitinib derivatives generated using MolAI resulted in 54 novel molecules where 6 of them demonstrated a docking score better than -10 kcal mol^{-1}. The three values below each compound represent, from left to right, the weights of the latent vectors for the molecules Top1 (ω_1), Top5 (ω_2), and Top7 (ω_3), respectively. The numbers in the parentheses indicate the corresponding Glide docking scores and free energy of binding values, respectively (both in kcal mol^{-1}).

(a)

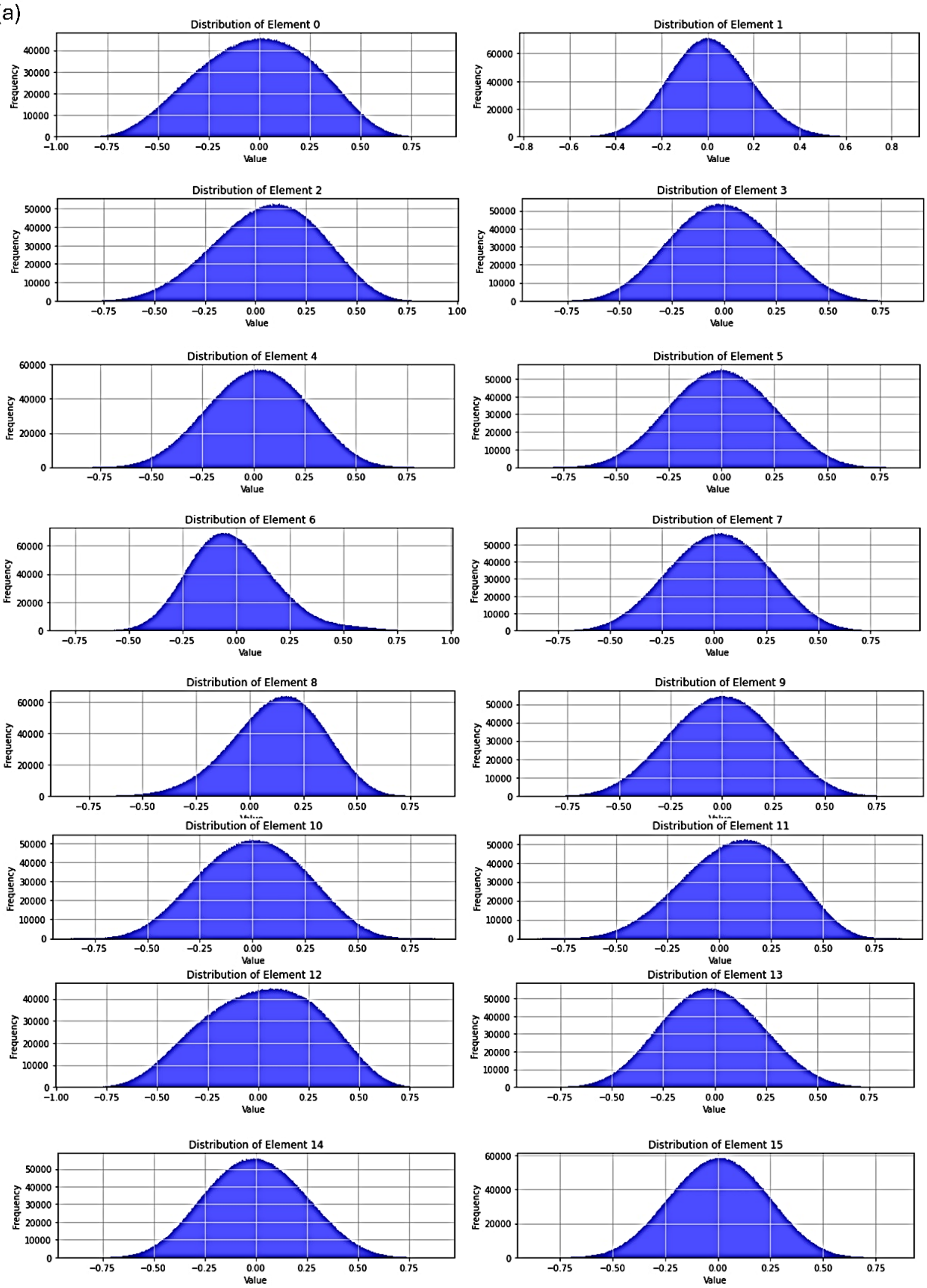
Distribution of Element 0
Distribution of Element 1
Distribution of Element 2
Distribution of Element 3
Distribution of Element 4
Distribution of Element 5
Distribution of Element 6
Distribution of Element 7
Distribution of Element 8
Distribution of Element 9
Distribution of Element 10
Distribution of Element 11
Distribution of Element 12
Distribution of Element 13
Distribution of Element 14
Distribution of Element 15
Frequency
Value

(b)

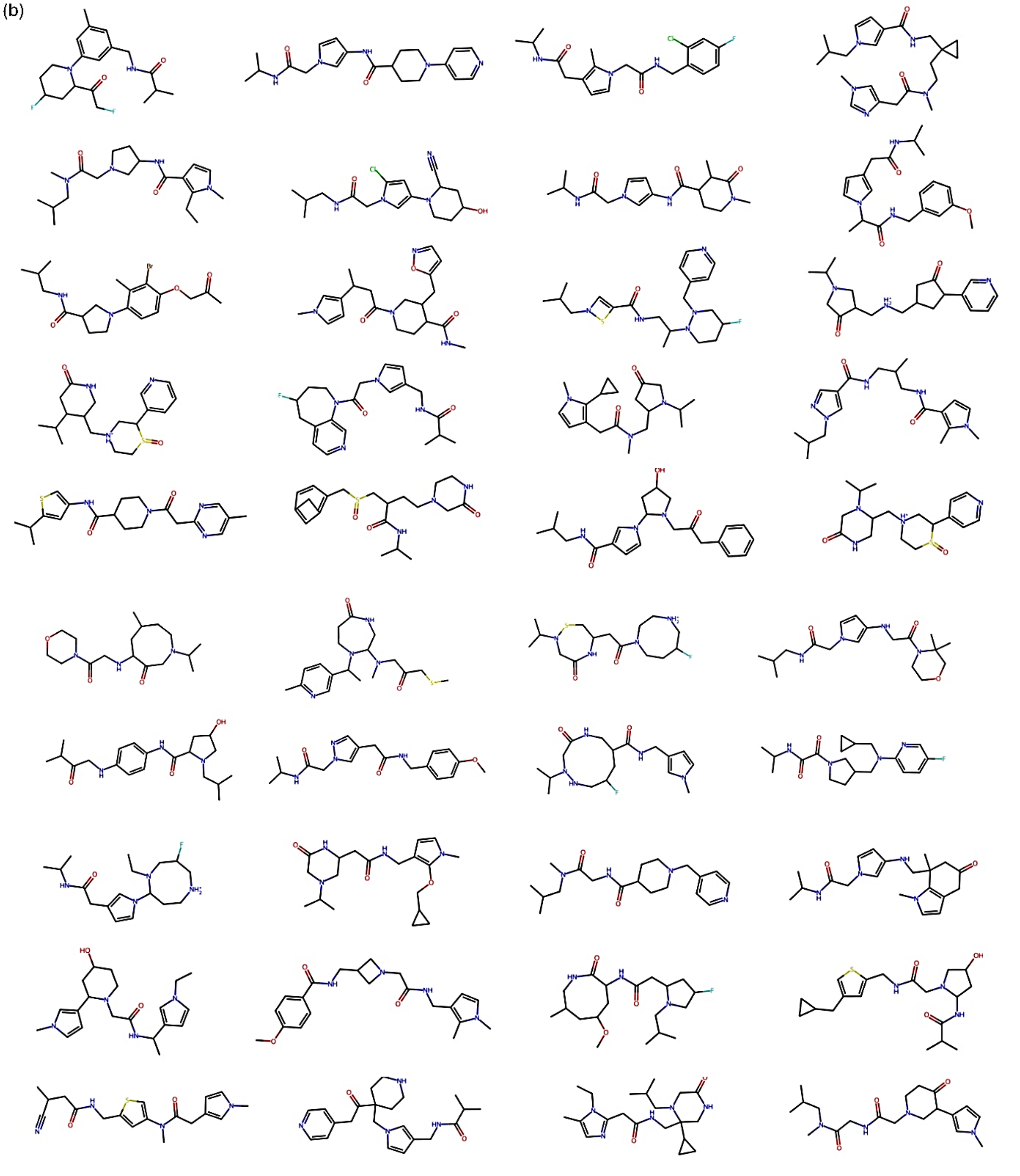

Fig. S3. (a) Distribution of the first 16 elements of latent vectors generated by MolAI for 1 million unique and valid molecules from the ZINC22 database. **(b)** Examples of *de novo* generated compounds resulting from random sampling based on the aforementioned latent space distributions.

Table S2. Pearson correlation coefficients and corresponding p-values for each molecular descriptor based on the optimal linear combinations of PCA components

Molecular descriptor	Pearson correlation coefficient	p-value
qed	0.9038	0.0000
MolWt	0.9994	0.0000
NumValenceElectrons	0.9997	0.0000
MaxPartialCharge	0.9683	0.0000
MinPartialCharge	0.9623	0.0000
BalabanJ	0.9374	0.0000
LabuteASA	0.9995	0.0000
TPSA	0.9976	0.0000
HeavyAtomCount	0.9997	0.0000
NumHAcceptors	0.9975	0.0000
NumHDonors	0.9972	0.0000
MolLogP	0.9993	0.0000
MolMR	0.9995	0.0000

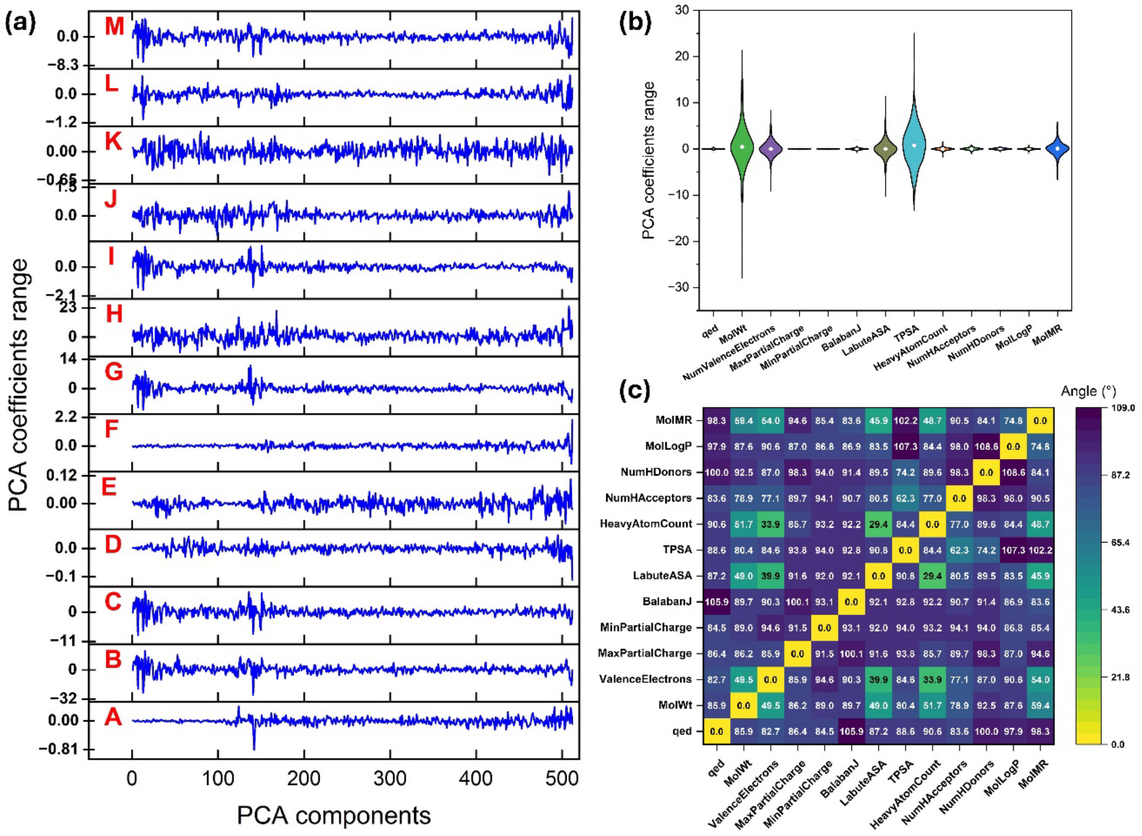

Fig. S4. The weights assigned to each PCA component **(a)** and their corresponding ranges for predicting the molecular descriptors **(b)**, as derived from the linear regression task. **(c)** Heatmap of the pairwise angles between descriptor vectors. In **(a)**, A-M stand for the molecular descriptors: qed, MolWt, NumValenceElectrons, MaxPartialCharge, MinPartialCharge, BalabanJ, LabuteASA, TPSA, HeavyAtomCount, NumHAcceptors, NumHDonors, MolLogP, and MolMR, respectively.

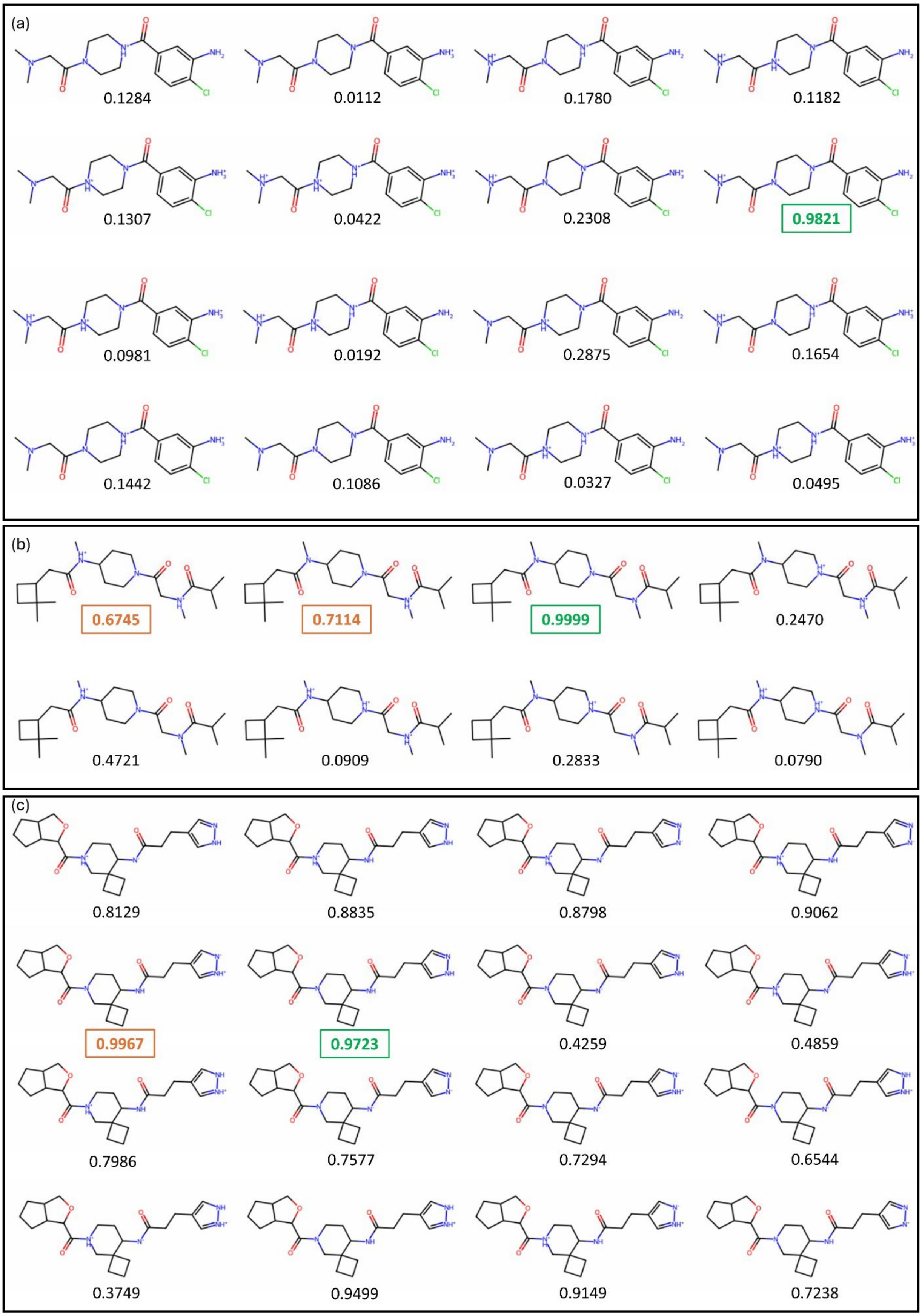

Fig. S5. (a) 16 different protonation states of a molecule from the Enamine test set generated by the Dimorphite DL tool (*Ref 43 in main text*), along with the iLP-calculated probability for each

protonation state being in class 1. The iLP model predicts the correct protonation state (highlighted in green) with a significantly higher probability compared to other incorrect protonation states. **(b)** Multiple protonation states classified as class 1 (probability > 0.5, highlighted in orange) but the correct protonation state can still be identified as the one with the highest probability. **(c)** In most unsuccessful cases, the correct protonation state is often the one with the second highest probability (highlighted in green).

Table S3. Fourteen datasets used for benchmarking MolAI-driven molecular descriptors through the training of the iADMET framework for ADMET features predictions.

Dataset	Acronym	Task	Datapoints before pre-processing	Datapoints after pre-processing	Definition of class 1
**Cell Effective Permeability**	*caco2*	Regression	906	865	-
**Lipophilicity**	*lipo*	Regression	4200	4174	-
**Solubility**	*sol*	Regression	9982	8217	-
**Bioavailability**	*bioav**	Classification	640	966	active
**Blood-Brain Barrier Perm.**	*bbb**	Classification	1975	2774	permeable
**Plasma Protein Binding Ratio**	*ppbr**	Classification	1614	1944	> 90%
**Half Life**	*hl**	Classification	667	1000	>24h
**CYP P450 2C19**	*cyp2c19*	Classification	12665	11982	inhibition
**CYP P450 2D6**	*cyp2d6**	Classification	13130	19942	inhibition
**CYP P450 3A4**	*cyp3a4*	Classification	12328	11630	inhibition
**CYP P450 1A2**	*cyp1a2*	Classification	12579	11884	inhibition
**CYP P450 2C9**	*cyp2c9**	Classification	12092	13712	inhibition
**Ames Mutagenicity**	*ames*	Classification	7255	6717	mutagenic
**hERG Central Blockers**	*herg**	Classification	306893	199914	blocker

**The SMOTE method was utilized to handle class imbalance*

Table S4. Summary of the iADMET framework performance metrics using MolAI-driven descriptors for regression and classification datasets on the validation sets using 5-fold cross validation

Dataset	R[a]	MAE[b]	Accuracy	Precision	Recall	F1-score	KAPPA	MCC	ROC-AUC	PR-AUC
caco2	**0.833** ±0.04	**0.340** ±0.03	-	-	-	-	-	-	-	-
lipo	**0.813** ±0.02	**0.516** ±0.02	-	-	-	-	-	-	-	-
sol	**0.901** ±0.01	**0.640** ±0.01	-	-	-	-	-	-	-	-
bioav	-	-	**0.919** ±0.011	**0.935** ±0.006	**0.900±** 0.027	**0.917** ±0.016	**0.837** ±0.023	**0.838** ±0.022	**0.95**	**0.91**
bbb	-	-	**0.948** ±0.012	**0.957** ±0.002	**0.938±** 0.023	**0.947** ±0.012	**0.896** ±0.024	**0.896** ±0.024	**0.98**	**0.97**
ppbr	-	-	**0.858** ±0.018	**0.892** ±0.030	**0.816±** 0.057	**0.850** ±0.026	**0.715** ±0.037	**0.720** ±0.033	**0.92**	**0.89**
hl	-	-	**0.973** ±0.012	**0.959** ±0.024	**0.988±** 0.004	**0.973** ±0.013	**0.946** ±0.025	**0.947** ±0.024	**0.98**	**0.94**
cyp2c19	-	-	**0.799** ±0.011	**0.781** ±0.009	**0.798±** 0.035	**0.789** ±0.015	**0.597** ±0.022	**0.597** ±0.022	**0.87**	**0.83**
cyp2d6	-	-	**0.925** ±0.001	**0.924** ±0.009	**0.926±** 0.012	**0.925** ±0.002	**0.850** ±0.002	**0.850** ±0.002	**0.97**	**0.97**
cyp3a4	-	-	**0.733** ±0.013	**0.708** ±0.032	**0.801±** 0.028	**0.751** ±0.008	**0.545** ±0.023	**0.550** ±0.021	**0.86**	**0.81**
cyp1a2	-	-	**0.833** ±0.005	**0.818** ±0.014	**0.836±** 0.030	**0.856** ±0.008	**0.705** ±0.018	**0.707** ±0.017	**0.92**	**0.90**
cyp2c9	-	-	**0.852** ±0.009	**0.836** ±0.0027	**0.879±** 0.041	**0.714** ±0.020	**0.565** ±0.025	**0.566** ±0.025	**0.87**	**0.76**
ames	-	-	**0.778** ±0.020	**0.808** ±0.022	**0.772±** 0.021	**0.790** ±0.019	**0.554** ±0.040	**0.555** ±0.040	**0.86**	**0.86**
herg	-	-	**0.956** ±0.005	**0.951** ±0.010	**0.963±** 0.010	**0.957** ±0.005	**0.913** ±0.009	**0.913** ±0.009	**0.99**	**0.99**

[a]*Pearson correlation coefficient*
[b]*mean absolute error*

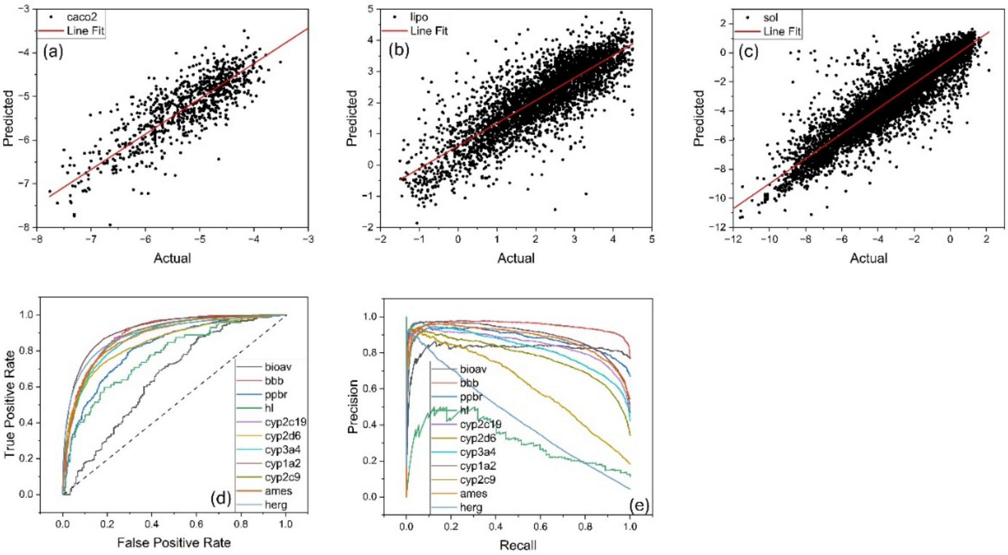

Fig. S6. ADMET feature predictions based on ChemBERTa descriptors. Scatter plots and best linear fit of the actual and predicted values for **(a)** caco2, **(b)** lipo, and **(c)** sol regression datasets. **(d)** ROC plots, **(e)** PR plots, and **(f)** confusion matrices for the classification datasets.

Table S5. Summary of the iADMET framework performance metrics using ChemBERTa-77M-MTR descriptors for regression and classification datasets on the validation sets using 5-fold cross validation

Dataset	R[a]	MAE[b]	Accuracy	Precision	Recall	F1-score	KAPPA	MCC	ROC-AUC	PR-AUC
caco2	**0.806** ±0.04	**0.366** ±0.04	-	-	-	-	-	-	-	-
lipo	**0.832** ±0.03	**0.485** ±0.01	-	-	-	-	-	-	-	-
sol	**0.912** ±0.01	**0.633** ±0.02	-	-	-	-	-	-	-	-
bioav	-	-	**0.798** ±0.041	**0.814** ±0.031	**0.956±** 0.042	**0.879** ±0.031	**0.255** ±0.093	**0.312** ±0.102	**0.65**	**0.82**
bbb	-	-	**0.882** ±0.019	**0.902** ±0.021	**0.952±** 0.006	**0.926** ±0.011	**0.636** ±0.062	**0.643** ±0.059	**0.89**	**0.95**
ppbr	-	-	**0.776** ±0.012	**0.794** ±0.018	**0.902±** 0.041	**0.843** ±0.015	**0.443** ±0.058	**0.462** ±0.038	**0.81**	**0.87**
hl	-	-	**0.893** ±0.029	**0.278** ±0.034	**0.132±** 0.017	**0.178** ±0.023	**0.146** ±0.021	**0.155** ±0.023	**0.77**	**0.31**
cyp2c19	-	-	**0.813** ±0.008	**0.793** ±0.012	**0.809±** 0.011	**0.801** ±0.005	**0.624** ±0.015	**0.624** ±0.015	**0.88**	**0.84**
cyp2d6	-	-	**0.868** ±0.003	**0.715** ±0.031	**0.458±** 0.019	**0.557** ±0.009	**0.484** ±0.008	**0.501** ±0.009	**0.84**	**0.62**
cyp3a4	-	-	**0.794** ±0.010	**0.756** ±0.008	**0.757±** 0.030	**0.756** ±0.015	**0.578** ±0.022	**0.578** ±0.022	**0.87**	**0.82**
cyp1a2	-	-	**0.840** ±0.005	**0.821** ±0.010	**0.843±** 0.015	**0.832** ±0.005	**0.679** ±0.010	**0.680** ±0.010	**0.92**	**0.90**
cyp2c9	-	-	**0.814** ±0.005	**0.726** ±0.020	**0.733±** 0.027	**0.729** ±0.015	**0.587** ±0.016	**0.587** ±0.016	**0.88**	**0.78**
ames	-	-	**0.800** ±0.018	**0.824** ±0.026	**0.808±** 0.019	**0.816** ±0.014	**0.598** ±0.038	**0.598** ±0.039	**0.87**	**0.88**
herg	-	-	**0.961** ±0.001	**0.663** ±0.038	**0.254±** 0.053	**0.363** ±0.049	**0.347** ±0.047	**0.392** ±0.030	**0.89**	**0.45**
